# Inflammatory Markers in Cancer Immunotherapy

**DOI:** 10.3390/biology10040325

**Published:** 2021-04-13

**Authors:** Deepak Ravindranathan, Viraj A. Master, Mehmet Asim Bilen

**Affiliations:** 1 Department of Hematology and Medical Oncology, Winship Cancer Institute of Emory University, Atlanta, GA 30322, USA; dravind@emory.edu; 2 Department of Urology, Emory University School of Medicine, Atlanta, GA 30322, USA; vmaster@emory.edu

**Keywords:** immunotherapy, inflammation, PD-1 inhibitors, PDL-1 inhibitors, CTLA-4 monoclonal antibodies, neutrophil-lymphocyte ratio, platelet-lymphocyte ratio, lymphocyte-monocyte ratio

## Abstract

**Simple Summary:**

Inflammation has been recognized to be linked to tumor development. Several markers of inflammation can be detected via blood such as variety of blood cells, which can be readily and easily obtained. These markers have been studied as ways to predict and prognosticate tumor response to chemotherapy. With the development of immunotherapy, namely immune checkpoint inhibitors (ICIs) such as cytotoxic T-lymphocyte-associated protein 4 (CTLA-4), programmed cell death protein 1 (PD-1), and programmed death ligand 1 (PDL-1) PDL-1 inhibitors, several markers have also been studied in assessing tumor response. In this review, we will discuss the various inflammatory markers that have been studied in several tumors treated with ICIs.

**Abstract:**

Chronic inflammation is considered a major risk factor for cancer formation. Inflammation within the tumor environment plays a role in its response to therapy, growth, and prognosis. Cancer associated inflammation is known to occur in the tumor microenvironment and in the systemic circulation, and is correlated with disease progression and prognosis in many cancers. Blood cells such as neutrophils, lymphocytes, platelets, and circulating proteins such as C-reactive protein, and interleukins, such as IL-6, have been associated with inflammatory responses, which contribute to tumorigenesis. Cancer has found ways to evade the immune response; a pathway that can attenuate the innate immune response is via blocking immune checkpoints. Development of monoclonal antibodies against inhibitory immune checkpoints such as cytotoxic T-lymphocyte-associated protein 4 (CTLA-4) and programmed cell death protein 1 (PD-1) have given rise to immunotherapy, which has shown remarkable responses in anti-tumor activity resulting in several U.S. Federal and Drug Administration (FDA)-approved checkpoint inhibitors. Various inflammatory markers and their prognostic and predictive implications in malignancies treated with immunotherapy will be discussed in this review.

## 1. Introduction

Chronic inflammation has been well-accepted to play a considerable role in carcinogenesis [1]. In fact, this relationship has been explored since the 19th century when Virchow found that there are leukocytes in tumor tissues and proposed a potential relationship between tumor and inflammation [2]. About a quarter of cancer cases may be due to infection and chronic inflammation [3]. The tumor microenvironment (TME) and immune system play a role in the occurrence and development of malignancies. Neutrophils act as effectors of both innate immunity and cell signaling in adaptive immune response and inhibit the activity of cytotoxic T lymphocytes in vitro. These also secrete tumor growth factors, cytokines, chemokines, such as TGF-beta, vascular endothelial growth factor (VEGF), IL-6, IL-8, IL-12, and matrix metalloproteinases which induce angiogenesis, supporting tumor growth [4,5]. Tumor cells also release granulocyte colony-stimulating factor which can increase the number of neutrophils. Monocytes differentiate into macrophages or dendritic cells in the tissue microenvironment. Platelets contribute to inflammation by releasing VEGF, which mediates the migration and extravasation of leukocytes, and platelet-derived growth factor (PDGF). T-lymphocytes in the TME have also been associated with improved clinical outcomes in patients affected by malignancies. T-lymphocytes can recognize and kill tumor cells which can affect proliferation and thereby further spread of disease.

Immune checkpoint inhibitors (ICIs), which are a form of immunotherapy, have been approved by the U.S. Federal and Drug Administration (FDA) as a treatment option for a variety of malignancies due to their durable clinical benefit in terms of treatment response and relatively favorable toxicity profile for patients.

In 2011, ipilimumab, which targets cytotoxic T-lymphocyte-associated protein 4 (CTLA-4), was approved by the U.S. FDA for the treatment of cancer after demonstrating improved overall survival (OS) in patients with metastatic melanoma [6,7]. Eventually, other ICIs such as pembrolizumab resulted in longer OS compared to ipilimumab in metastatic melanoma. In advanced non-small cell lung cancer (NSCLC), pembrolizumab also fared better and demonstrated better progression free survival (PFS) and OS compared to platinum-based chemotherapy [8].

As of 2020, a total of seven immuno-oncology (IO) agents have been approved, including ipilimumab, which targets CTLA-4, atezolizumab, avelumab, and durvalumab, which target programmed cell death ligand 1 (PD-L1), and nivolumab, pembrolizumab, and cemiplimab, which target programmed cell death 1 (PD-1). These agents have been approved for numerous types of cancer such as melanoma, NSCLC, head and neck squamous cell malignancies, liver, urothelial, renal cell, gastric, breast, and colorectal cancers. Additional IO agents are currently under investigation [7], and Table 1 lists the currently approved ICI agents.

Considerable effort has been made towards identifying biomarkers of response to immunotherapy as predictive and prognostic markers since the use of these IO agents has increased in the past few years in oncology. Only 15–60% patients respond as expected to ICIs and can experience immune related adverse events [9]. Identifying biomarkers for this particular patient population is crucial.

Reduction in lymphocyte count can decrease anti-tumor response and affect ICI effectiveness as ICIs rely on the inhibitory signal function of T lymphocytes. Increased lymphocyte infiltration in the TME is associated with better prognosis and response to immunotherapy [10,11]. Given this, neutrophil-to-lymphocyte (NLR), platelet-to-lymphocyte ratio (PLR), and monocyte-to-lymphocyte ratio (MLR) have been used as inflammatory markers to predict outcomes in various malignancies. Measurement of these cells is simple and conveniently conducted on a complete blood count with differential from blood. This review will discuss each inflammatory ratio and its validation in the treatment of tumors with immunotherapy. 

## 2. Neutrophil-to-Lymphocyte Ratio (NLR)

NLR has been utilized as an inflammatory marker for prognosis, disease recurrence and response to treatment [12]. Elevated NLR has been unfortunately associated with poorer prognosis in numerous tumors [10,11,12,13,14,15,16,17]. Templeton et al. performed a systematic review and meta-analysis of 100 studies which found that high NLR was associated with poor OS in all tumor subtypes in solid oncology [5]. For example, in patients with metastatic renal cell carcinoma (mRCC) and tumor thrombus undergoing cytoreductive nephrectomy, a NLR < 4.0 had longer median survival versus those patients with NLR > 4.0 and was proposed to use to predict survival after cytoreductive surgery in mRCC [18]. In fact, the first discussion of NLR was in 1976 by Russell et al. who described the immunologic constituents in tumor foci of sarcoma in mice at various time points after inoculating sarcoma. In that study, intratumoral immune cells were studied and a high number of T-lymphocytes was seen in regressing tumors. In progressing tumors, a higher number of neutrophils was seen [19].

In patients with metastatic melanoma treated with ipilimumab, it has been reported that a NLR ≥ 5 is associated with inferior PFS and OS. Given this, it was proposed that a lower NLR suggests that those particular patients will benefit from ipilimumab in the context that T cells are more stimulated as evidenced by a higher ratio of neutrophils to lymphocytes [20]. Bilen et al. studied 90 patients with advanced malignancies receiving IO-based treatments in a phase I clinical trial and found that high baseline NLR, MLR, and PLR were associated with worse OS and PFS (*p* < 0.05) and lower chance of benefit (NLR and PLR; *p* < 0.05). After treatment, increased NLR, MLR, and PLR six weeks after baseline were also associated with shorter OS and PFS (*p* < 0.052) [21]. 

Another study reported that patients with mRCC treated with PD-1/PD-L1 inhibitors had a higher baseline NLR which was associated with lower objective response rate (ORR), shorter PFS, and shorter OS. Six weeks after the start of therapy, higher NLR was a stronger predictor of ORR, PFS, and OS than baseline NLR was. Relative change in NLR by >25% from baseline to six weeks after ICI therapy was associated with reduced ORR and was an independent prognostic factor for PFS and OS; however, a decrease in NLR by >25% was associated with improved ORR, PFS, and OS [22].

Bilen et al. reported that in patients with mRCC treated with nivolumab, a baseline NLR of <5.5 prior to nivolumab start was associated with superior PFS and OS [15]. Jeyakumar et al. reported that NLR greater than 4 predicated shorter OS and PFS in 57 patients receiving ICI for genitourinary cancers [23]. Nakaya et al. reported that NLR at two and four weeks after initiation of nivolumab might serve as a useful marker for treatment response of disease progression in advanced NSCLC. Specifically, an NLR of <3 before treatment was associated with longer median PFS and NLR > 3 was associated with shorter median PFS [24].

Fujita et al. conducted a retrospective study of 20 patients with advanced gastric cancer and at a cut off for NLR of 2, patients with NLR > 2 had prolonged OS and PFS compared to the group of patients with NLR < 2 [25]. Ota et al. studied institutional data of 98 patients who received nivolumab and found that poor prognostic factors of OS were pretreatment NLR of >3 and NLR difference of >2 over 60 days before and after receiving nivolumab. Those with NLR difference less than 2 had longer median OS [26].

With a review of 509 patients, Li et al. reported the trend of NLR at baseline and first month of treatment in patients receiving ICI for metastatic disease. Via this, they evaluated the prognostic value of baseline and changes in NLR from baseline to being treated with ICI. Patients with NLR of less than 5 had significantly longer OS (*p* < 0.001) and the change in NLR was shown to be a predictor of OS and non-linear in nature. Those who had moderate decrease in NLR had the longest survival whereas significant decrease or increase in NLR was associated with shorter survival [27].

The majority of the studies looking at this ratio involve either nivolumab or pembrolizumab. Katayama studied NLR in 81 patients diagnosed with NSCLC who received atezolizumab as monotherapy and found that patients with high NLR at baseline exhibited shorter PFS and OS compared to those with a low NLR [28]. Other ratios such as LMR and PLR were also studied in this study and these particular ratios will be discussed in following sections.

## 3. Platelet-to-Lymphocyte Ratio (PLR)

Attention has also been directed looking at the role of PLR as a predictive marker for response to immunotherapy. VEGF augments angiogenesis by increasing vascular permeability and PDGF is frequently upregulated in tumors and support stroma, and tumor proliferation by stimulating the process of angiogenesis [29]. Increased platelets can further support tumor progression by increasing angiogenesis to the tumor and producing adhesion molecules [30,31]. Lymphocytes also play a role in anti-tumor immunity and release cytokines that confer anti-tumor immunity [32]. Elevated PLR has been associated with poor prognosis in variety of cancer patients. Zer et al. found that with a cutoff value of 200, there was no significant relationship between baseline PLR and efficacy of immunotherapy [33]. In one retrospective study with 187 patients with NSCLC, PLR below 200 was associated with longer PFS (*p* = 0.028) and OS (*p* = 0.001), and higher ORR (*p* = 0.04) [34].

A meta-analysis of 1845 patients with NSCLC from 21 studies that included treatment with three ICIs found that high NLR was associated with poor OS and PFS. Similarly, elevated PLR was associated with inferior OS and PFS. However, in subgroup analysis, there was no correlation between post-treatment PLR and survival outcomes [35]. Another meta-analysis by Xu et al. reviewed 12 eligible studies that included 1430 patients with cancer and reported that an high PLR negatively affected the efficacy of ICIs; high PLR in patients was seen with a shorter OS compared to patients with low PLR (HR = 2.02, 95%CI: 1.27 to 2.38, *p* = 0.0006). In the subgroup analyses, the prognostic role of PLR on OS and PFS was dependent on certain factors such as cancer type, region, and cutoff value. For patients with metastatic NSCLC, stage of disease, the ICI agent, or the number of lines of treatment did not seem to influence the prognostic role of this ratio [36]. Diem et al. also reported that elevated PLR was associated with shorter OS and PFS and lower response rates in patient diagnosed with metastatic NSCLC treated with nivolumab [14].

Interestingly, Shabto et al. reported a novel risk stratification system for urothelial carcinoma patients treated with ICI using platelet-to-lymphocyte risk as an inflammatory marker in addition to Eastern Cooperative Oncology performance status (ECOG PS), presence of liver metastasis, and albumin [37].High PLR was seen to be demonstrating shorter PFS and OS compared to patients with low PLR with NSCLC treated with atezolizumab [28]. Higher PLR of >168.13 before the fifth dose of ICIs was noted to be a prognostic marker and correlated with smaller OS in patients with NSCLC receiving nivolumab and durvalumab [38].

## 4. Lymphocyte-to-Monocyte Ratio (LMR)

Similar to other markers, LMR has been studied as a potential biomarker. A less than optimal number of lymphocytes can lead to an insufficient immunologic reaction to the tumor, thus promoting tumor progression and metastasis [39]. Monocytes infiltrate tumors and differentiate into tumor-associated macrophages, which are involved in tumor proliferation, invasion, metastasis, neovascularization, and recurrence. Hence, an increased level of monocytes can indicate high tumor burden. Therefore, LMR can reflect the degree of host immunity and degree of tumor progression. A low lymphocyte count and high monocyte count means poor anti-tumor immunity and elevated tumor burden and is thus associated with poor prognosis [40]. In a meta-analysis by Wang et al, by reviewing 20 studies which included 8304 patients, low LMR was seen in patients with metastatic NSCLC with shorter OS (HR = 1.63; 95% CI: 1.44–1.85, *p* < 0.0001) and PFS (HR = 1.49, 95% CI: 1.25–1.77, *p* < 0.001) [41].

With regards to immunotherapy, in a cohort of 87 patients with advanced NSCLC who received nivolumab, an increase of at least 10% in LMR at four weeks after initiation of nivolumab relative to pretreatment values was correlative with a positive ORR. With a cutoff value of >10%, increase in LMR was associated with longer PFS and OS. Increase in LMR was associated significantly with higher ORR and prolonged PFS [42]. Failing et al. looked at the LMR ratio for the first time in patients with metastatic melanoma treated with pembrolizumab and found that pretreatment LMR of at least 1.7 showed improved PFS and OS [43]. Rebuzzi et al. reported preliminary results of the Meet-URO 15 study, which was to evaluate the prognostic role of certain markers in metastatic renal cell cancer treated with nivolumab. In a sample size of 150 patients, LMR > 3 was correlated with significantly longer OS but not PFS as well as similar overall response rate and disease control rate (DCR) compared to when the LMR < 3. From this preliminary study, the authors concluded that the prognostic role of LMR is uncertain in this setting [44]. Low LMR was seen to be associated with shorter PFS and OS compared to patients with high LMR with NSCLC treated with atezolizumab [28].

## 5. Monocyte-to-Lymphocyte Ratio (MLR)

The inverse of LMR, MLR, has been studied as well. A study by Martini et al. looked at 100 patients with mRCC treated with immunotherapy and MLR was reported as a stronger, more effective predictor of survival and used in risk score calculation to stratify as good, intermediate, poor, or very poor risk. In fact, good risk in this model was set with MLR of being less than <0.93 [45]. As mentioned before, in the study in which Bilen et al. studied 90 patients with advanced malignancies receiving IO-based treatments in a phase I clinical trial, notably, high baseline MLR, along with NLR and PLR, was associated with worse OS and PFS (*p* < 0.05) but not with a lower chance of benefit like NLR and PLR. After treatment, increased MLR in addition to NLR and PLR six weeks after baseline were also associated with shorter OS and PFS (*p* < 0.052) [21]. Recently, Kadano et al. looked at this ratio in patients with advanced urothelial carcinoma treated with pembrolizumab. From 91 patients in this study, pretreatment NLR and the change in one month in the NLR were associated with OS after patients were treated with pembrolizumab. Pretreatment NLR < 2.9 and change of the NLR of less than 43% in a month was associated with longer OS than the pretreatment NLR being >2.9 and one month change of being greater than or equal to 43% [46]. This ratio is not as heavily studied compared to the other ratios discussed in this review. 

## 6. C-Reactive Protein (CRP)

CRP is another serum inflammatory marker that has been studied in numerous infections and used as a biomarker in cancer. Elevated CRP levels have been shown to be associated with increased risk of cancer [47]. In addition, elevated CRP levels have been shown to also associate with cancer progression and decreased survival [48,49,50,51]. Harris et al. formally assessed CRP as a time-dependent prognostic variable for OS in patients treated with targeted therapy for clear cell and non-clear cell mRCC and that time-dependent effects are seen as representation of the intensity of systemic inflammation which can serve as a prognostic biomarker for mRCC [52]. Both pre-treatment and indeed post treatment CRP levels can help prognosticate survival after intervention in various genitourinary malignancies [53]. In patients with localized RCC who underwent nephrectomy, a prospective study looked at preoperative and postoperative CRP levels and found that postoperative CRP is the better predictor of metastasis and mortality following surgical resection [54].

High levels of baseline CRP have been associated with poor response to chemotherapy in various malignancies. As an acute phase protein of hepatic origin, CRP reflects the process of systemic inflammation from cancer and its related complications such as cachexia, pyrexia, and fatigue. In addition, higher CRP levels have been correlated with low levels of CD4+ T-cells, which play a key role in the antitumor immune response facilitated by immunotherapy [48,49,50,51].

Riedl et al. showed that elevated pretreatment CRP levels are associated with poor outcomes. Increase in CRP over time is a strong indicator of an elevated progression risk and on the contrary, decline in CRP is associated with treatment response. In conclusion, this study shows that CRP may serve as a simple biomarker for assessing and monitoring ICI treatment benefit in advanced NSCLC patients [55]. In a study of 95 patients with advanced melanoma treated with ipilimumab, decreased levels of CRP by the end of treatment were associated with better disease control and increased OS [56]. Brown et al. reported a retrospective analysis utilizing modified Glasgow prognostic score (mGPS) in 78 patients with mRCC treated with immunotherapy. The mGPS was developed as a scoring system predictive of clinical outcomes across multiple malignancies and incorporates inflammatory markers albumin and CRP. It was reported that a higher mGPS at baseline was associated with worse OS, and at six weeks as well, and hence, higher CRP values contributed to higher mGPS [57]. The same group of authors also reported, using mGPS in 53 patients with metastatic urothelial cell carcinoma, that higher mGPS was again correlated with shorter OS and high correlation with other inflammatory biomarkers, such as NLR, PLR, and MLR. Again, high CRP levels was seen in these patients with high mGPS [58].

## 7. IL-6 Levels

Interleukin 6 (IL-6) has been shown to increase CRP levels and hence has also been explored as an inflammatory biomarker [59]. In clinical practice, serum IL-6 levels are usually applied to inflammatory or infectious diseases. Increased IL-6 levels have been reported in patients diagnosed with breast, cervical, esophageal, head and neck, ovarian, pancreatic, prostate, and renal cancers. Elevated IL-6 levels have been associated with increased incidence of adverse events in patients receiving immunotherapy [60]. Laino et al. studied patients from the CheckMate 064, 066, and 067 studies and found that high levels of IL-6 were associated with worse response and shorter survival in patients with melanoma treated with nivolumab. Serum IL-6 and CRP may therefore function as prognostic factors in melanoma patients receiving single agent and combination checkpoint inhibition [61]. 

Among 125 NSCLC patients, the ORR and disease control rate (DCR) were higher in patients with low IL-6 (<13.1 pg/mL) than in those with high IL-6. The median PFS was 6.3 months in the low IL-6 group, significantly longer than the 1.9 months in the high IL-6 group. The median OS in the low IL-6 group was significantly longer than in the high IL-6 group (not reached vs. 7.4 months, 95% CI, 4.8–10.0). Thus, baseline serum IL-6 levels could be a potential biomarker for predicting the efficacy and survival benefit of PD-1/PD-L1 inhibitors in NSCLC [62].

Keegan et al. measured plasma cytokines via an ultrasensitive single-molecule array assays in patients with NSCLC before and during treatment with PD-1 inhibitor. From 46 patients, decrease in levels was associated with longer PFS and degree of change in IL-6 differed between best overall response and correlated with changes in C reactive protein levels [63].

## 8. Body Mass Index (BMI): Increased Body Mass as a Production of Inflammation

BMI has been explored as a predictor of clinical outcomes in patients with metastatic disease; however, no conclusive results have been found. One retrospective study did report an association between BMI and clinical outcomes among patients with metastatic melanoma treated with either targeted therapy or immunotherapy [64].

Another study found that overweight patients with metastatic melanoma with loss of muscle mass experienced adverse effects earlier in the course while receiving immunotherapy [65]. Richtig et al. reported that overweight patients with melanoma (76 total) treated with ipilimumab had higher response rate and longer OS when compared to patients who were not overweight [66]. Moreover, another study by Wang et al. also reported that obese (BMI > 30) patients with advanced cancer had longer PFS and OS when treated with ICIs [67]. 

A retrospective study by Cortellini et al. of about 1000 patients with advanced cancer treated with anti-PD-1/PD-L1 inhibitors reported a significant association between being overweight (BMI > 25) and clinical response to immunotherapy when given as first-line therapy. ORR was significantly higher in overweight compared to non-overweight patients. Median PFS and OS were both longer as well [68].

Giorgi et al. looked at the role of systemic inflammation in patients with RCC treated with nivolumab and showed that normal BMI combined with higher systemic inflammation tripled the risk of death and was thought to be a prognostic factor for OS [69]. Interestingly, Eun et al. reported that patients with higher BMI and receiving more cycles of pembrolizumab were seen to have an increased risk of immune related adverse events [70].

It has been postulated that white adipose tissue has a role in the host defense system as it is a source of cytokines and chemokines. Adipose tissue modulates the Th1/Th2 balance, increases pro-inflammatory macrophages, and activates T-cells, hence harnessing the mechanism of immunotherapy, which may explain improved outcomes with immunotherapy in those who are overweight. Gelibter et al. commented that obesity should not be seen as a positive prognostic factor but as rather a contributor to immune dysfunction and tumor progression that can be treated with PD-1/PD-L1 inhibition [71].

Sarcopenia, or decreased levels of skeletal muscle mass and function, can be defined as skeletal mass index (SMI) more than two SDs below the mean for healthy adults (male patients, <55 cm^2^/m^2^; female patients, <39 cm^2^/m^2^). Martini et al. investigated the effect of inflammation and sarcopenia on outcomes of patients with solid tumors treated with immunotherapy participating in phase I clinical trials. A four-level stratification system was created in which low-risk was defined as having PLR < 242 and being nonsarcopenic, intermediate-risk as having PLR < 242 and being sarcopenic, high-risk with PLR > 242 and being nonsarcopenic and very-high-risk with PLR > 242 and being sarcopenic. Out of a cohort of 90 patients, very high-risk, high-risk, and intermediate-risk patients had statistically significant shorter overall survival and progression free survival compared with low risk patients [72].

Akce et al. looked at sarcopenia and its association in patients with metastatic hepatocellular carcinoma patients treated with anti PD-1 antibody therapy. In this retrospective study, sarcopenia was sex-specific and was calculated based on skeletal muscle density derived using SliceOmatic software, which assesses computed tomography and magnetic resonance images at mid-L3 level. The skeletal muscle density was then converted to skeletal muscle index (SMI) by dividing it by height (m^2^). Sex-specific sarcopenia was defined by the median value of SMI and cut-off for males and females was SMI of 43 and 39, respectively. However, sex-specific sarcopenia did not seem to predict OS or PFS and it was proposed that perhaps baseline BMI and NLR may predict OS in advanced HCC patient treated with immunotherapy [73].

**Figure 1:**
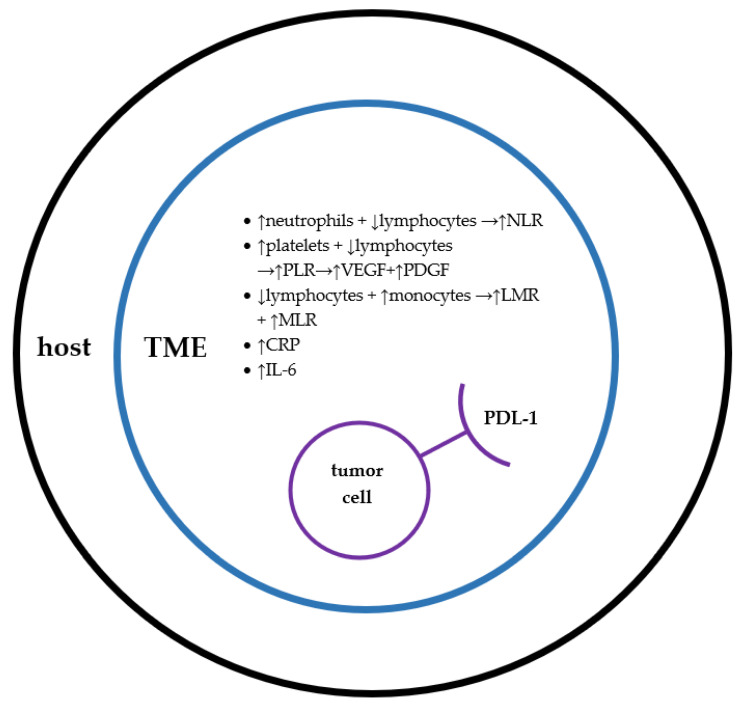
Interaction of various factors in host, tumor microenvironment (TME) that leads to tumor cell proliferation.

Figure 1 summarizes the interaction of various inflammatory markers discussed in this review leading to tumor progression.

## 9. Conclusions

ICIs have emerged as a modality of treatment for advanced malignancies. Discovery of inhibition of negative immune regulation led to American immunologist, Dr. James P. Allison and Dr. Tasuku Honjo, immunologist from Japan to receive the Nobel Prize in Physiology or Medicine in 2018. Current indications for immunotherapy are based on PDL-1 percentage detected on tumor cells or based on combined positive score (CPS). FDA approval for immunotherapy has been primarily in the metastatic setting and there are clinical trials are studying its role in the neoadjuvant and adjuvant settings. Hence, identifying and developing biomarkers that can predict responses to cancer immunotherapy is vital. PD-L1 immunohistochemical expression is studied well as a biomarker in ICIs and in fact has been approved by the FDA. However, the inflammatory markers can be easily detected via blood via common laboratory assays at numerous medical centers in the world. In this review, we have discussed in detail a variety of inflammatory markers such as NLR, PLR, LMR, MLR, CRP, and IL-6 which can be obtained in peripheral blood and their prognostication in response to immunotherapy. Many studies look at baseline ratios prior to treatment and more and more studies are emerging that are looking at changes throughout treatment. As more approvals for immunotherapy agents continue to happen, further studies of inflammatory markers are recommended and hopefully there could be widely used prognostic models in the future.

## Figures and Tables

**Table 1:** List of currently approved ICIs.

Name	Target	Year of Approval	Malignancies Approved for
Atezolizumab	PD-L1	2016	urothelial carcinoma
		2020	non-small cell lung cancer
Avelumab	PD-L1	2017	Merkel cell carcinoma
		2019	renal cell carcinoma
		2020	urothelial carcinoma
Durvalumab	PD-L1	2017	urothelial carcinoma
		2018	non-small cell lung cancer
Cemiplimab	PD-1	2018	cutaneous squamous cell carcinoma
		2021	basal cell carcinoma
		2021	non-small cell lung cancer
Ipilimumab	CTLA-4	2011	melanoma
		2018	renal cell carcinoma
		2018	MSI-H/dMMR colorectal cancer
Pembrolizumab	PD-1	2014	melanoma
		2015	non-small cell lung cancer
		2016	head and neck cancer
		2017	microsatellite instability-high/mismatch repair solid tumors
		2017	gastric cancer
		2018	Hodgkin’s lymphoma
		2018	urothelial carcinoma
		2018	cervical cancer
		2018	hepatocellular carcinoma
		2018	Merkel cell carcinoma
		2019	renal cell carcinoma
		2019	small cell lung cancer
		2019	esophageal carcinoma
		2019	endometrial cancer
Nivolumab	PD-1	2014	melanoma
		2015	non-small cell lung cancer
		2015	renal cell carcinoma
		2016	Hodgkin’s lymphoma
		2016	head and neck cancer
		2017	urothelial carcinoma
		2017	microsatellite instability-high/mismatch repair solid tumors
		2017	hepatocellular carcinoma
		2018	small cell lung cancer

## Data Availability

Not applicable.
